# Targeting services to reduce social inequalities in utilisation: an analysis of breast cancer screening in New South Wales

**DOI:** 10.1186/1743-8462-4-12

**Published:** 2007-06-05

**Authors:** Stephen Birch, Marion Haas, Elizabeth Savage, Kees Van Gool

**Affiliations:** 1 Centre for Health Economics Research and Evaluation, University of Technology Sydney, PO Box 123 Broadway, Sydney, Australia; 2 Centre for Health Economics and Policy Analysis, McMaster University, 1200, Main Street West, Hamilton, Ontario L8N 3Z5, Canada

## Abstract

**Background:**

Many jurisdictions have used public funding of health care to reduce or remove price at the point of delivery of services. Whilst this reduces an important barrier to accessing care, it does nothing to discriminate between groups considered to have greater or fewer needs. In this paper, we consider whether active targeted recruitment, in addition to offering a 'free' service, is associated with a reduction in social inequalities in self-reported utilization of the breast screening services in NSW, Australia.

**Methods:**

Using the 1997 and 1998 NSW Health Surveys we estimated probit models on the probability of having had a screening mammogram in the last two years for all women aged 40–79. The models examined the relative importance of socio-economic and geographic factors in predicting screening behaviour in three different needs groups – where needs were defined on the basis of a woman's age.

**Results:**

We find that women in higher socio-economic groups are more likely to have been screened than those in lower groups for all age groups. However, the socio-economic effect is significantly less among women who were in the actively targeted age group.

**Conclusion:**

This indicates that recruitment and follow-up was associated with a modest reduction in social inequalities in utilisation although significant income differences remain.

## Background

Policy makers concerned with maximising health gain within given resource constraints will aim to deliver health care to those who are most able to benefit. Consequently, any systematic variations in utilisation within a subgroup of the population with similar needs and similar capacity to benefit from health care would represent an inefficient allocation of resources [1].

Given that the distribution of needs for health care within populations is generally inversely related to the ability to pay for care, many jurisdictions have used public funding of health care to reduce or remove price at the point of delivery of services [2]. Removing price at the point of delivery essentially removes one barrier to access but is not necessarily a sufficient condition to ensure health care utilisation is in line with health care need. For example, the opportunity cost of traveling to (and waiting at) the point of delivery may be greater among poorer groups and hence inversely related to needs. As a result, utilisation will be determined by (non-price) demand factors, which determine the opportunity cost (or shadow price) of utilisation, as opposed to needs (or risk) factors that determine the efficiency of resource utilisation from a societal or policy-makers perspective. Existing research indicates that systematic variations in health care use remain even after controlling for needs/risks and that this feature is common to many publicly funded health care systems [3-7]. In other words, removing the price barrier to accessing care may increase utilisation among all groups, irrespective of their relative needs for care while needs-based differences in utilisation between rich and poor remain [2].

An alternative approach is to introduce policies that encourage or support increased utilisation among target-age groups (ie those considered most in need of the care) within a universal system of full public funding. However, because active recruitment programs employ substantial resources it is important to establish whether they are effective in reducing non-needs based differences in utilisation and hence contribute to an improved allocation of health care resources. Although there is a considerable literature on factors associated with the levels of utilization in screening programs covering both general populations and target-age groups [8-17], little attention has been directed at identifying the effect of program features on access to and/or utilization of screening services.

The focus of the study is the 'BreastScreen' program in New South Wales, Australia which provides screening free at the point of delivery for all women aged 40 and over. Under the program, need for screening is defined by age with 50–69 year old women being targeted as the priority population for screening and the focus of a systematic call and recall system. We set out to test whether this systematic approach to encouraging utilization results in lower levels of inequalities in self-reported screening than in the rest of the female population eligible for screening. In addition, because the non-price elements of opportunity cost may differ between geographic communities, because of differences in access costs, we also consider whether the observed social inequalities in self-reported utilisation vary among different regions covered by the program.

## Background

Breast cancer is the most common cancer and a leading cause of death among women in many countries [18]. In Australia one in every ten women will develop the disease and over 2500 women die from breast cancer each year [19]. Although the mortality rate from breast cancer has steadily declined over the last decade, the incidence of breast cancer continues to rise, indicating that the decline in mortality is due to earlier detection and/or more effective treatment once detected.

BreastScreen NSW is a state based screening program funded by both the Australian and NSW governments and is part of a national mammography program introduced in 1990 [20]. Under the program, mammography is offered at over 500 locations nationwide via a mixture of fixed, relocatable and mobile screening units. All women aged 40 and over are eligible to be screened every two years free at the point of delivery. No referral by a physician is required but a woman's general practitioner is provided with the results of the screen and is informed of any further services required. In the case of New South Wales (NSW), the Area Health Services (AHSs) have been charged with the responsibility for the management and delivery of the program. At the time this study was conducted, NSW consisted of 17 AHS covering both densely populated metropolitan populations and more scattered rural and remote populations.

The program aimed to increase coverage of breast screening among all women in the target-age group (50–69) to 70% and targeted these women to receive promotional materials and recruitment strategies, including letters of invitation, encouraging them to attend for screening. The program also aimed to "ensure equitable access for women aged 50–69 years to the program" [20]. Although access was not defined explicitly, the implication is that utilisation should not be systematically related to any social, economic or cultural factors. Any systematic variation in use would indicate that the equitable access objective had not been satisfied. In this way, the policy for this age group was needs-based with the intention that all women in this age group be screened because all were deemed to be 'target-age'. From an epidemiological perspective, important variations in risk may occur within the target-age group related to hereditary or other factors. However, the program was not concerned with identifying and implementing priority groups within this target age group. Instead, the program's objectives and policies imply that each woman in the age group is deemed to be of equal priority.

For women aged 40–49 or 70 and above, BreastScreen NSW also offers free services at the point of delivery. During the period covering this research, all women aged 40–49 were re-invited by letter for routine re-screening once they had attended the BreastScreen program. However, the program did not actively recruit women in these age groups to initiate screening. Indeed, the program aimed to constrain screening to a maximum of 40% of women age 40–49 and 15% of women 70–79 [21]. Whilst no explicit policy levers were used to discourage screening in these groups, the program was not concerned with increasing screening, or with ensuring that screening was performed equally across all groups of women in these age groups. As a result, utilisation in these age groups is predominantly 'demand driven' with each woman's perception of benefits and opportunity costs determining whether or not to attend for screening.

The configuration of the program in this way provides an opportunity to evaluate the impact of 'needs-based' strategies for increasing utilisation on social inequalities in utilisation by comparing utilisation patterns where use is 'needs-driven' (the target-age group) with those where use is 'demand-driven' (the non target-age group) and testing for variation in screening rates between AHSs. Our analysis is organised around three research questions:

1. Does socio-economic status explain variation in reported use of breast screening?

2. How important Is socio-economic status in explaining inequalities in screening for target-age groups compared with inequalities in screening for non target-age groups?

3. Does the association between utilisation and socio-economic status vary among geographically-defined populations?

## Methods

### Analytical model

An individual will choose to screen if utility is greater with screening than without. Let *V*_1_* be the difference between utility with screening and without. This difference is not observed, but is assumed to arise from the model

V1∗=X′β+μ
 MathType@MTEF@5@5@+=feaafiart1ev1aaatCvAUfKttLearuWrP9MDH5MBPbIqV92AaeXatLxBI9gBaebbnrfifHhDYfgasaacH8akY=wiFfYdH8Gipec8Eeeu0xXdbba9frFj0=OqFfea0dXdd9vqai=hGuQ8kuc9pgc9s8qqaq=dirpe0xb9q8qiLsFr0=vr0=vr0dc8meaabaqaciaacaGaaeqabaqabeGadaaakeaacqWGwbGvdaqhaaWcbaGaeGymaedabaGaey4fIOcaaOGaeyypa0JafmiwaGLbauaaiiGacqWFYoGycqGHRaWkcqWF8oqBaaa@367D@

where μ has a normal distribution with mean zero and variance one. What is observed is whether an individual screens or not, that is

S={1if V∗>00if V∗≤0
 MathType@MTEF@5@5@+=feaafiart1ev1aaatCvAUfKttLearuWrP9MDH5MBPbIqV92AaeXatLxBI9gBaebbnrfifHhDYfgasaacH8akY=wiFfYdH8Gipec8Eeeu0xXdbba9frFj0=OqFfea0dXdd9vqai=hGuQ8kuc9pgc9s8qqaq=dirpe0xb9q8qiLsFr0=vr0=vr0dc8meaabaqaciaacaGaaeqabaqabeGadaaakeaacqWGtbWucqGH9aqpdaGabeqaauaabeqaciaaaeaacqaIXaqmaeaacqWGPbqAcqWGMbGzcqqGGaaicqWGwbGvdaahaaWcbeqaaiabgEHiQaaakiabg6da+iabicdaWaqaaiabicdaWaqaaiabdMgaPjabdAgaMjabbccaGiabdAfawnaaCaaaleqabaGaey4fIOcaaOGaeyizImQaeGimaadaaaGaay5Eaaaaaa@4227@

This gives rise to the probit model

Prob[*S *= 1] = Φ(*X'β*)

where Φ(·) denotes the cumulative distribution of the standard normal function and *X *denotes the explanatory variables.

An estimated probit coefficient (*β*) indicates how a unit change in the explanatory variable will impact on the probit index measured in units of standard deviations. The results are more easily interpreted in terms of marginal effects. For continuous explanatory variables, the marginal effect indicates the impact on the probability of being screened associated with a unit difference in the explanatory variable, when other variables are set to their baseline values. Where categorical data are entered using simple 1–0 indicator variables, the marginal effect is the difference in probability of screening between an individual with the characteristic and without it (i.e., with the baseline characteristic) with all other variables set to their baseline values.

### Data

The 1997 and 1998 NSW Health Surveys were the main sources of data [22]. We pooled the survey responses which were obtained via a telephone-administered questionnaire conducted on a random sample of NSW residents aged 16 or over. To generate a representative sample of the population as well as take account of the population distribution across AHS, the survey was based on a sample of around 2000 residents in each of the 17 AHS, weighted to take account of household size, the age and sex distribution of the population. It was completed by approximately 34,000 residents, representing 0.36% of the adult population. Survey questions covered a wide range of topics relating to health and illness, health risks and health care utilisation together with background information relating to social and demographic characteristics.

The dependent variable for the analysis was the self-report of having had a mammogram in the last two years. Respondents were also asked the reason for their most recent mammogram. Using the responses to this question, we excluded women whose utilisation of mammograms was for diagnostic rather than primary screening purposes.

Traditional models of health care utilisation group explanatory variables as need, predisposing, enabling and system variables [23,24]. For the population under consideration, women age 40–79, need was measured as a 1–0 variable with the target-age group, women aged 50–69, being in greater need and in line with the BreastScreen program's stated objectives. Predisposing variables were country of birth and aboriginal status. Socio-economic status was used as an enabling variable and AHS of residence was introduced as a proxy for a system variable (see Table 1 for variable definitions).

**Table 1:** Variables and definitions used in the study

Variable	Definition
Screening mammography	At least one mammogram obtained for screening purposes (ie not diagnostic) in the last 2 years
Target-age women	Women aged 50–69 years (the target age range for the BreastScreen program)
Non target-age women (young)	Women aged 40–49 years
Non target-age women (older)	Women aged 70–79 years
Income	Average personal income per week ('00)
Income squared	Average personal income per week squared
Australian-born	Women who were born in Australia
Aboriginal (ATSI)	Women who identify as being of Aboriginal or Torres Strait descent

The 1997 and 1998 NSW Health Surveys contained a number of variables that could be used to approximate socio-economic status but they did not contain personal income data. To overcome this problem we used a Monte Carlo Data Augmentation technique that imputes income based on matching each individual in the study population with individuals in an additional (third) dataset [25]; the Household Expenditure Survey (HES) 1998/99, a dataset that does contain data on income [26]. Individuals were matched on the basis of three socioeconomic status variables (in receipt of any government pension or benefit, covered by private health insurance and highest education level achieved) as well as age and sex. Although in principle it would be helpful to match on other variables, such as family size, marital status and employment status, these variables were not available in both data sets.

Each individual in the NSW Health Survey was randomly assigned an income from the pool of matched observations in the HES. Both income and income squared were entered in the model to allow for non-linearity in the association between utilisation and income.

### Analysis

We estimated a probit model for the probability of having had a mammogram in the last two years for the entire sample combined (i.e. all women aged 40–79 excluding women reporting having had a mammogram for diagnostic purposes) including dummy variables for the non target-age groups as additional explanatory variables (Model 1). The equation was then re-estimated for each age group separately (i.e., the target-age group, aged 50–69, and two non target-age groups eligible for screening under the programme, aged 40–49 and 70–79) (Model 2). Each of the equations was then re-estimated with the AHS indicator variables included (Model 3). The specific research questions were addressed as follows:

#### Does socio-economic status explain variation in reported use of breast screening?

This question was considered by estimating the marginal effect of income on the probability of screening for the whole survey sample. The null hypothesis was that the impact of income is not significantly different from zero. Rejection of the null would indicate that socio-economic status is an important determinant of the probability of reporting having a screening mammogram.

#### How important is socio-economic status in explainingvariations in screening for the target-age group compared with variations in screening for non target-age groups?

This question was considered by comparing the estimated marginal effect of income for the target-age group with the corresponding values for the non target-age groups. The null hypothesis was that the estimated impact of income in the target-age group is equal to or greater than the estimated coefficients on income in the non target-age groups. Rejection of the null would indicate that the attempt to promote utilisation among target-age groups has led to greater equity in mammography utilisation.

#### Does the association between utilisation and socio-economic status vary among communities?

This question was considered by introducing regional information into the analysis. Details of the AHS where the woman lived were introduced into the equation as an explanatory variable. Models allowing interactions between AHS and socio-economic status were then estimated to test for variation in the association between income and utilisation among AHS. A likelihood ratio test was used to determine whether allowing interactions led to an improvement in the model. The null hypothesis was that the additions of AHS and interaction terms do not improve the model. Rejection of the null hypothesis would indicate that socio-economic status is an important determinant of the probability of reporting having a screening mammogram and that this importance varies depending on where a woman lives.

## Results

Table 2 records the sample size, mean and standard deviation for each variable separately for the target-age and non target-age groups. Mean imputed income was inversely related to age, as was the proportion of women of Aboriginal and Torres Strait Islander (ATSI) background. The proportion of Australian born women was positively correlated with age. Self-reported screening, however, shows no clear linear age pattern, but is highest in the target-age group.

**Table 2:** Means and standard deviations of variables

	**All** **(age 40–79)**		**High risk** **(age 50–69)**		**Non High Risk** **(age 40–49)**		**Non High Risk** **(age 70–79)**	
	Mean	StDev	Mean	StDev	Mean	StDev	Mean	StDev

Screening rate	57.8%	49.4%	73.8%	44.0%	37.1%	48.3%	53.4%	49.9%
Income ($'00/wk)	$3.26	$1.54	$2.79	$1.19	$4.56	$1.48	$2.11	$0.43
Australian born	79.5%	40.4%	79.5%	40.4%	77.5%	41.7%	83.0%	37.6%
Aboriginal	1.4%	11.6%	1.2%	10.9%	1.8%	13.5%	0.8%	9.1%

Observations	11308		5482		3785		2041	

The coefficients in the probit model, once transformed to marginal effects, estimate the impact of the explanatory variable on the probability of utilisation compared to the base case. For model 1 the base is target-age, Australian-born, non-aboriginal with imputed income equal to the mean imputed income level of all women in the sample. For model 2 the base is Australian-born, non-aboriginal with imputed income equal to the mean imputed income level of women in target-age sub-samples. For model 3 the base is Australian-born, non-aboriginal, living in the Northern Sydney AHS with imputed income equal to the mean AHS imputed income level of women in target-age and non target-age sub-samples. The estimated coefficients and marginal effects for models 1 and 2 are presented in Table 3 and for model 3 in Table 4. Coefficients and marginal effects significant at the 5% level are indicated; standard errors for the marginal effects were obtained by bootstrapping.

**Table 3:** Probit results and marginal effects for Models 1 and 2

	**Model 1**		**Model 2**					
		
	**All (age 40 – 79)**		**High Risk (age 50 – 69)**		**Non High Risk (age 40 – 49)**		**Non High Risk (age 70 – 79)**	
		
	Coefficient	Marginal effect	Coefficient	Marginal effect	Coefficient	Marginal effect	Coefficient	Marginal effect
		
Dummy age 40–49	-1.010 *	-0.373 #	-	-	-	-	-	-
Dummy age 70–79	-0.473 *	-0.161 #	-	-	-	-	-	-
Income ($'00/wk)	0.248 *	0.021 #	0.350 *	0.023 #	0.398 *	0.013 #	1.517	0.118
Income squared	-0.027 *		-0.048 *		-0.040 *		-0.288	
Non Australian born	-0.174 *	-0.054 #	-0.286 *	-0.091 #	-0.079	-0.031 #	0.021	-0.008
Aboriginal	-0.196	-0.123 #	-0.234	-0.177	-0.145	-0.085	-0.207	-0.091
Constant	0.265 *	-	0.213	-	-1.171 *	-	-1.713	
		
Log *L*	-6,980.4		-3,059.1		-2,514.5		-1,389.5	
Observations	11,308		5,482		3,785		2,041	
Pseudo R^2^	0.091		0.014		0.005		0.0073	

Notes:

* Coefficient significant at the 5% levelM

# Marginal effect sigificant at the 5% level

**Table 4:** Marginal effect on the probability of screening of an extra $100 per week by level of weekly income and age group

Group (average $/wk)	$100/wk	$200/wk	$300/wk	$400/wk
All ($326)	6.9%	4.5%	2.6%	0.9%
Low 40–49 ($456)	9.1%	8.2%	6.0%	3.1%
High ($279)	8.9%	4.9%	1.7%	-1.0%
Low 70–79 ($211)	33.4%	14.4%	-8.1%	-30.4%

In general, the signs on the estimated coefficients in Table 3 show that non target-age, non-Australian born and aboriginal women were less likely, but higher-income women were more likely to report being screened. In model 2, being born outside Australia significantly reduced the probability of screening by 9.1% for the target-age group and 3.1% for the younger non target-age group. Aboriginality reduced the screening probability significantly only in model 1 (by 12.3%). However, the observable effect of aboriginal status was limited by a small sample size, which may explain why the effect diminished in Model 2.

### Does socioeconomic status explain variation in reported use of breast screening?

In terms of the full sample (Table 3, Model 1), women in the non target-age groups were less likely to have been screened. Those aged 40 to 49 were 37.3% and those aged 70 to 79 were 16.1% less likely to have had a screen than the target-age group. Women with higher imputed incomes were more likely to have had a screen. Imputed income remained a significant factor even after controlling for the other variables. The positive coefficient on imputed income and negative coefficient on the square of imputed income indicates that the effect of imputed income on screening diminishes with higher imputed incomes. The marginal effect of imputed income therefore depends on the level of imputed income in the baseline. In model 1, at the mean imputed income of the entire sample ($326 per week) an extra $100 increased the probability of screening by 2.1%. The association between imputed income and probability of screening remained after entering the AHS indicator variables (see Table 5). In other words, this 'imputed income-use' relationship could not be explained by clustering of different imputed income groups among AHS. Hence, the null hypothesis that the estimated coefficient on imputed income is not different from zero was rejected.

**Table 5:** Probit results and marginal effects for Model 3

	**High Risk (age 50 – 69)**		**Non High Risk (age 40 – 49)**		**Non High Risk (age 70 – 79)**	
	Coefficient	Marginal effect	Coefficient	Marginal effect	Coefficient	Marginal effect

Income ($'00/wk)	0.344 *	0.015 #	0.404 *	0.012	1.466	0.104 #
Income squared	-0.050 *		-0.041 *		-0.282	-0.007
Non Australian born	-0.268 *	-0.069 #	-0.045	-0.018	-0.019	-0.087
Aboriginal	-0.200	-0.131 #	-0.152	-0.076	-0.204	
Central Sydney	-0.233	-0.059 #	-0.004	-0.001	-0.253	-0.099
South Eastern Sydney	-0.271 *	-0.070 #	0.026	0.010	0.081	0.030
South Western Sydney	-0.503 *	-0.143 #	-0.170	-0.066	-0.208	-0.081
Wentworth	-0.412 *	-0.113 #	-0.027	-0.011	0.066	0.025
Western Sydney	-0.291 *	-0.076 #	-0.179	-0.069	-0.237	-0.092
Central Coast	-0.183	-0.045	-0.079	-0.031	0.029	0.011
Far West	-0.305 *	-0.080 #	0.074	0.029	-0.159	-0.061
Greater Murray	-0.234 *	-0.059 #	-0.048	-0.019	-0.297	-0.116 #
Macquarie	-0.543 *	-0.157 #	-0.194	-0.075	-0.239	-0.093
Mid North Coast	-0.227 *	-0.057 #	0.170	0.067	-0.147	-0.057
Mid Western	-0.658 *	-0.197 #	-0.108	-0.042	-0.423 *	-0.166 #
New England	-0.239 *	-0.057 #	-0.299 *	-0.012	-0.058	-0.101
Northern Rivers	-0.225 *	-0.061 #	-0.030	-0.113 #	-0.259	-0.022
Southern	-0.254 *	-0.065 #	-0.269 *	-0.103 #	-0.349 *	-0.137 #
Hunter	-0.086	-0.020	0.354 *	0.140 #	0.061	0.023
Illawarra	-0.334 *	-0.088 #	-0.173	-0.067	-0.424 *	-0.167 #
Constant	0.500 *		-1.148 *		-1.510	

Log *L*	-3022.030		-2484.780		-1372.700	
Observations	5482		3785		2041	
Pseudo R^2^	0.026		0.017		0.019	

### How important is socio-economic status in explaining variations in screening in the target-age group compared with variations in screening in non target-age groups?

The marginal effect of imputed income differs across age groups as shown in the results for model 2 in Table 3. For the target-age group, an additional $100 of imputed weekly income significantly increases the probability of screening by 2.3%. This result is slightly higher than for the full sample (model 1) while for the younger non target-age group, the impact is smaller (1.3%). However, these comparisons are problematic because mean imputed income differs among the three age groups. To overcome this problem we calculated the marginal effect of a $100 increase in imputed weekly income on the probability of screening for different levels of imputed weekly income (Table 4). For each group, the combined effect of the imputed income and imputed income-squared terms produces a diminishing marginal imputed income effect, i.e., the effect of an additional $100 of imputed weekly income on probability of screening diminishes as imputed weekly income increases. Moreover, the rate of reduction of the marginal effect of imputed income is greatest for the older non target-age group and least for the younger non target-age group.

Although imputed income is a factor in explaining differences in screening in the target-age group, it is less important in explaining differences in screening in this group than it is among non target-age groups. Targeting may therefore have helped to reduce inequalities in screening among women from groups with different imputed incomes. At higher imputed income levels, the marginal effect of imputed income becomes negative for both the target-age group and the older non target-age group (i.e. probability of screening falls as imputed income rises) and the size of the effect is greater among the older group. This might be due to few individuals falling in these high imputed income categories in these groups and hence the estimates of marginal effects being unreliable outside the range of the data.

### Does the association between utilisation and socio-economic status vary among communities?

The results following the introduction of *AHS of residence *variable into the model are presented in Table 5. The likelihood ratio tests indicated that introducing this variable significantly improves the model's precision. The part of the null hypothesis that suggested there would be no improvement was therefore rejected. However in the case of interactions between area and imputed income, only in the case of the older age group was the model (marginally) improved. In other words, there was no evidence that the association between imputed income and utilisation among target-age women varied across areas.

Location did have an important direct effect on screening independently of imputed income. Target-age women in all areas were less likely to have had a screen than women in North Sydney. This finding, which might be explained by area differences in access to services, was significant for all but two areas. In the non target-age groups, the findings were much less consistent both in terms of the signs (greater or less than North Sydney) and significance of the estimated coefficients. In other words, the effect of location is largely confined to target-age women, i.e., the subjects of targeting.

## Discussion

The dependent variable for the analysis was women's self-reported breast screening behaviour in the last two years. The validity of data from the survey is dependent on the accuracy of respondents' self reports, in this case, how accurately women aged 40–79 answered questions about whether they had had a mammogram in the previous two years. Others may be due to women having a "diagnostic" mammogram on referral from their GP, but understanding this to be a "screening" mammogram.

Although the data from the NSW Health Survey are now eight years old, more recent reports indicate that the utilization of the screening program has not changed substantially since then (ref needed here). Administrative data on mammography utilisation are available from the BreastScreen program. These data are limited to the AHS level and hence cannot be linked to individual patient characteristics.

Comparison of the two data sets found that the incidence of self-reported utilisation of mammography over the previous two years was 9.5% higher than administrative data on utilisation recorded under the BreastScreen program. After excluding the responses from women who stated that they had a mammogram for diagnostic rather than primary screening purposes, this difference was reduced but still evident. This difference introduces a potential bias into the study if women in particular age or socioeconomic groups or regions systematically under or over report their screening behaviour. Some of these differences may be due to women aged 70 and over continuing to screen if they have reached this age group after being targeted for screening ie a "flow-on" effect of screening. During this period some BreastScreen services in NSW re-invited women up to 75 years. In areas where women were not re-invited, they could still attend if they rang for an appointment (Ann Brassil, personal communication, March 2007). Other differences may be due to women having a "diagnostic" mammogram on referral from their GP, but understanding this to be a "screening" mammogram.

The use of imputed income represents a potential limitation for the study. Whilst we used conventional techniques to impute income [25], in the absence of any income questions in the NSW Health Survey we were unable to establish the validity of our imputation of incomes. Moreover, there was only a limited range of variables in the survey that represented reasonable socio-economic 'dimensions' for estimating imputed income. Nevertheless self-reported utilization in the entire sample was found to increase with imputed income, as would be expected for actual income.

Recruitment and follow-up strategies, such as those employed by BreastScreen NSW, do not affect the opportunity cost of utilising the service. Instead, they inform, emphasise and remind women about the importance and timeliness of using the system already in place and are aimed at changing the perception or expectation of benefits associated with service utilisation. As a result, if the opportunity costs of using the system differ by social group, other things being equal, increasing the perceived benefits of mammography screening would be expected to lead to a greater response among those groups of the population for whom the opportunity cost of utilisation is lowest. We might expect that higher-income groups face lower opportunity costs and therefore recruitment and follow-up strategies would have a greater impact on utilisation among higher- income groups than among lower-income groups, other things being equal.

The results presented in this paper show that among target-age women, those in higher imputed income groups are more likely to have been screened than those in lower imputed income groups. However this income-related difference is less than that observed in non target-age women. Moreover, the greater imputed income effect in non target-age women cannot be attributed to age-related factors since it occurs in both non target-age groups (i.e., both younger and older age groups than the target-age women). Unfortunately no population-based data on self-reported utilization of screening are available for the period before the Breast Screen program. However there is no a priori reason why income-based differences in utilization should have varied systematically between age groups. The age groups under consideration only became meaningful from an analytical perspective once the screening program defined separate target-age and non target-age groups (which, in turn, was based on evidence from trials of higher relative cost-effectiveness of screening for women aged 50–69). As a result, our findings suggest that the observed smaller income-based differences in utilization observed in the target-age group are associated with the strategy of recruitment and follow-up used for target-age women. This is indicative of some improvements in equity of access and hence a modest improvement in the allocation of program resources relative to the program's objectives.

These findings appear to be inconsistent with the notion that the additional features of the BreastScreen program did little if anything to address the opportunity costs of breast screening. There are a number of possible reasons for this finding. It may be that opportunistically the BreastScreen facilities were located and organised within each AHS in a way that had greater impact on opportunity costs of lower-income women than higher-income women. Alternatively, the finding might reflect an underlying socio-economic difference in knowledge of breast cancer screening and the availability of services free at the point of delivery such that targeted messages were not needed among higher-income women but served as an effective trigger to action in lower-income groups.

The lower imputed income effect in utilisation amongst target-age women is insufficient to determine whether the program of targeting is associated with a reduction in social inequalities in self-reported utilization of the breast screening services. This would require comparing the value of these 'improvements' in distribution of services with the opportunity costs of the BreastScreen program.

Although target-age women exhibited a lower imputed income effect, there is also evidence of significant Area effects not observed in the non target-age groups. It seems unlikely that this is explained by differences in the effectiveness of targeting strategies between Areas since there was no evidence of interactions between Area and imputed income. Instead these Area effects might be explained by between-Area differences in the availability and accessibility of BreastScreen facilities. Accurate data on screening capacity across AHSs is not generally available. Although the number of screening sites per 10,000 eligible women, and the proportion of mobile sites are greater in AHSs that cover more rural and remote populations, these data do not cover the screening capacity of sites. This would be an important topic for future research. Nevertheless, the lower imputed income effect is observed after controlling for AHS and hence between AHS differences in availability cannot explain the estimated association between targeting particular age groups and the lower observed levels of inequalities in self-reported utilisation.

The apparent reduction in income effects associated with targeting strategies contrasts with the results from other research investigating programs aimed at reducing socio-economic inequalities in service utilisation and outcomes. For example, Reading et al [27] found that a population-based program aimed at improving childhood immunisation rates based on information sharing about non-immunised children and an immunization referral service was associated with an increase in disparities in immunization rates between deprived and affluent areas. Both Arblaster et al [28] and Birch [4] note the lack of evidence for programs aimed at reducing inequalities. Rather than measuring between-group differences in service utilisation, researchers have tended to focus attention on measuring change (in utilisation or health status) among particular subgroups of the population without considering the effect of the program on the population distribution [29].

This problem arises when rates of utilisation in the population are considered in isolation from the variation in factors that determine utilisation within the population. Although from an epidemiological or policy perspective women in the target-age age group might be considered as a homogeneous group, from a social and economic perspective they exhibit considerable heterogeneity.

Because it is precisely these social and economic factors that are likely to affect behaviour, we need to consider methods for increasing utilisation in the context of the prevailing distributions of the determinants of behaviour. Notwithstanding the modest effect of targeting estimated in this paper, if we want to increase utilisation rates among all groups of women in the target age-group then we need to better understand the current barriers to utilisation across the different subpopulations within this target group.

## Competing interests

The author(s) declare that they have no competing interests.

## Authors' contributions

SB conceived and participated in the design of the study, assisted in the interpretation and led the drafting of the manuscript.

MH conceived and participated in the design of the study, assisted in the interpretation and drafting of the manuscript.

ES designed and carried out the analysis of the data, assisted in the interpretation and drafting of the manuscript.

KvG conceived and participated in the design of the study, assisted in the analysis and interpretation of the results and contributed to the drafting of the manuscript.

All authors read and approved the final manuscript.

## References

[B1] O’Donnell O PC (1991). Equity and the distribution of UK National Health service resources.. Journal of Health Economics.

[B2] Birch S, Abelson J (1993). Is reasonable access what we want? Implications of, and challenges to, current Canadian policy on equity in health care.[see comment]. International Journal of Health Services.

[B3] Alter DA, Naylor CD, Austin P, Tu JV (1999). Effects of socioeconomic status on access to invasive cardiac procedures and on mortality after acute myocardial infarction. New England Journal of Medicine.

[B4] Birch S (1999). The 39 steps: the mystery of health inequalities in the UK.[see comment]. Health Economics.

[B5] Birch S, Eyles J, Newbold KB (1993). Equitable access to health care: methodological extensions to the analysis of physician utilization in Canada. Health Economics.

[B6] Birch S, Gafni A (2005). Achievements and challenges of medicare in Canada: Are we there yet? Are we on course?. International Journal of Health Services.

[B7] Black DM (1980). Inequilities in health: Report of a research working group.

[B8] Achat H, Close G, Taylor R (2005). Who has regular mammograms? Effects of knowledge, beliefs, socioeconomic status, and health-related factors. Preventive Medicine.

[B9] Ahmed NU, Fort JG, Elzey JD, Belay Y (2005). Empowering factors for regular mammography screening in under-served populations: pilot survey results in Tennessee.[see comment]. Ethnicity & Disease.

[B10] Bynum JP, Braunstein JB, Sharkey P, Haddad K, Wu AW (2005). The influence of health status, age, and race on screening mammography in elderly women. Archives of Internal Medicine.

[B11] Husaini BA, Emerson JS, Hull PC, Sherkat DE, Levine RS, Cain VA (2005). Rural-urban differences in breast cancer screening among African American women. Journal of Health Care for the Poor & Underserved.

[B12] Joslyn SA, Foote ML, Nasseri K, Coughlin SS, Howe HL (2005). Racial and ethnic disparities in breast cancer rates by age: NAACCR Breast Cancer Project. Breast Cancer Research & Treatment.

[B13] Kudadjie-Gyamfi E, Consedine N, Magai C, Gillespie M, Pierre-Louis J (2005). Breast self-examination practices among women from six ethnic groups and the influence of cancer worry. Breast Cancer Research & Treatment.

[B14] Organisation for Economic Co-operation and Development. (2003). A disease-based comparison of health systems : what is best and at what cost?.

[B15] Rosenberg L, Wise LA, Palmer JR, Horton NJ, Adams-Campbell LL (2005). A multilevel study of socioeconomic predictors of regular mammography use among African-American women. Cancer Epidemiology, Biomarkers & Prevention.

[B16] Shenson D. Bolen J. Adams M. Seeff L. Blackman D. (2005). Are older adults up-to-date with cancer screening and vaccinations?. Preventing Chronic Disease.

[B17] Tu SPJ (2005). Cancer prevention screening: a cross-border comparison of United States and Canadian Chinese women. Prev Med.

[B18] Yankaskas BC, Gill KS (2005). Diagnostic mammography performance and race: outcomes in Black and White women. Cancer.

[B19] Australian Institute of Health and Welfare., NHMRC National Breast Cancer Centre (Australia), Australasian Association of Cancer Registries. (1999). Breast cancer in Australian women, 1982-1996. Can ; 6.

[B20] Australian Institute of Health and Welfare., BreastScreen Australia., Australia. Dept. of Health and Aged Care. (2000). BreastScreen Australia : achievement report 1997 and 1998.

[B21] NSW Health and West Sydney Area Health Service (1999). BreastScreen NSW, Strategic Plan 1999-2004.

[B22] NSW Health (1999). NSW Health Survey Report.

[B23] Aday LA, Andersen R (1974). A framework for the study of access to medical care. Health Services Research.

[B24] Andersen R, Newman JF (1973). Societal and individual determinants of medical care utilization in the United States. Milbank Memorial Fund Quarterly - Health & Society.

[B25] Williamson P, Rees PMDWP (2002). Appendix to Chapter 17: item imputation. The census data system.

[B26] Australian Bureau of Statistics. (2001). Household expenditure survey, Australia, 1998-99.

[B27] Reading R, Colver A, Openshaw S, Jarvis S (1994). Do interventions that improve immunisation uptake also reduce social inequalities in uptake?. BMJ.

[B28] Arblaster L, Lambert M, Entwistle V, Forster M, Fullerton D, Sheldon T, Watt I (1996). A systematic review of the effectiveness of health service interventions aimed at reducing inequalities in health. Journal of Health Services & Research Policy.

[B29] Acheson DB (1998). Independent inquiry into inequilities in health:report.

